# The phosphorylation to acetylation/methylation cascade in transcriptional regulation: how kinases regulate transcriptional activities of DNA/histone-modifying enzymes

**DOI:** 10.1186/s13578-022-00821-7

**Published:** 2022-06-03

**Authors:** Pin Zhao, Samiullah Malik

**Affiliations:** 1 Guangdong Key Laboratory for Biomedical Measurements and Ultrasound Imaging, School of Biomedical Engineering, Shenzhen University Health Science Center, 518060 Shenzhen, China; 2 Department of Pathogen Biology, Shenzhen University Health Science Center, 518055 Shenzhen, China

**Keywords:** Phosphorylation, DNA/histone modifying-enzymes, Transcription factors, Histone acetylation, Methylation, Transcription activity

## Abstract

Transcription factors directly regulate gene expression by recognizing and binding to specific DNA sequences, involving the dynamic alterations of chromatin structure and the formation of a complex with different kinds of cofactors, like DNA/histone modifying-enzymes, chromatin remodeling factors, and cell cycle factors. Despite the significance of transcription factors, it remains unclear to determine how these cofactors are regulated to cooperate with transcription factors, especially DNA/histone modifying-enzymes. It has been known that DNA/histone modifying-enzymes are regulated by post-translational modifications. And the most common and important modification is phosphorylation. Even though various DNA/histone modifying-enzymes have been classified and partly explained how phosphorylated sites of these enzymes function characteristically in recent studies. It still needs to find out the relationship between phosphorylation of these enzymes and the diseases-associated transcriptional regulation. Here this review describes how phosphorylation affects the transcription activity of these enzymes and other functions, including protein stability, subcellular localization, binding to chromatin, and interaction with other proteins.

## Introduction

Protein phosphorylation is crucial for various cellular processes, including cell growth, DNA damage, metabolism, inflammation. By definition, various protein kinases phosphorylate serine (Ser), threonine, and tyrosine of targeted proteins. Phosphorylation is one of the most common post-translational modifications (PTMs). It generally alters the structural conformation and interaction of proteins, by which it directly influences protein stability, cellular localization, protein/DNA binding, and enzymatic activity [1].

Approximately two-thirds of 21,000 known human proteins have been reported to be phosphorated with over 200,000 specific protein sites, and over 760,000 additional sites are predicted to be phosphorylated in several websites, including the Cell Signaling Technology PhosphoSitePlus (www.phosphosite.org) and the Kinexus PhosphoNET (www.phosphonet.ca). Among these reported proteins, there are some transcriptional factors (TFs) and the cofactors. It has been clearly defined how TFs and the cofactors organize and regulate gene transcription. In general, TFs firstly occupy DNA elements in a sequence-specific manner, and then recruit the RNA polymerases to the gene’s promoter region, which simultaneously involve chromatin remodeling complexes and histone (de)acetyltransferases for promoter accessibility [2]. It is reported that about 1,600 human proteins have been identified as transcriptional factors [3]. TFs control gene expression and chromatin status by binding specific DNA sequences, which are thus tightly controlled in normal cells [2].

**Fig. 1:**
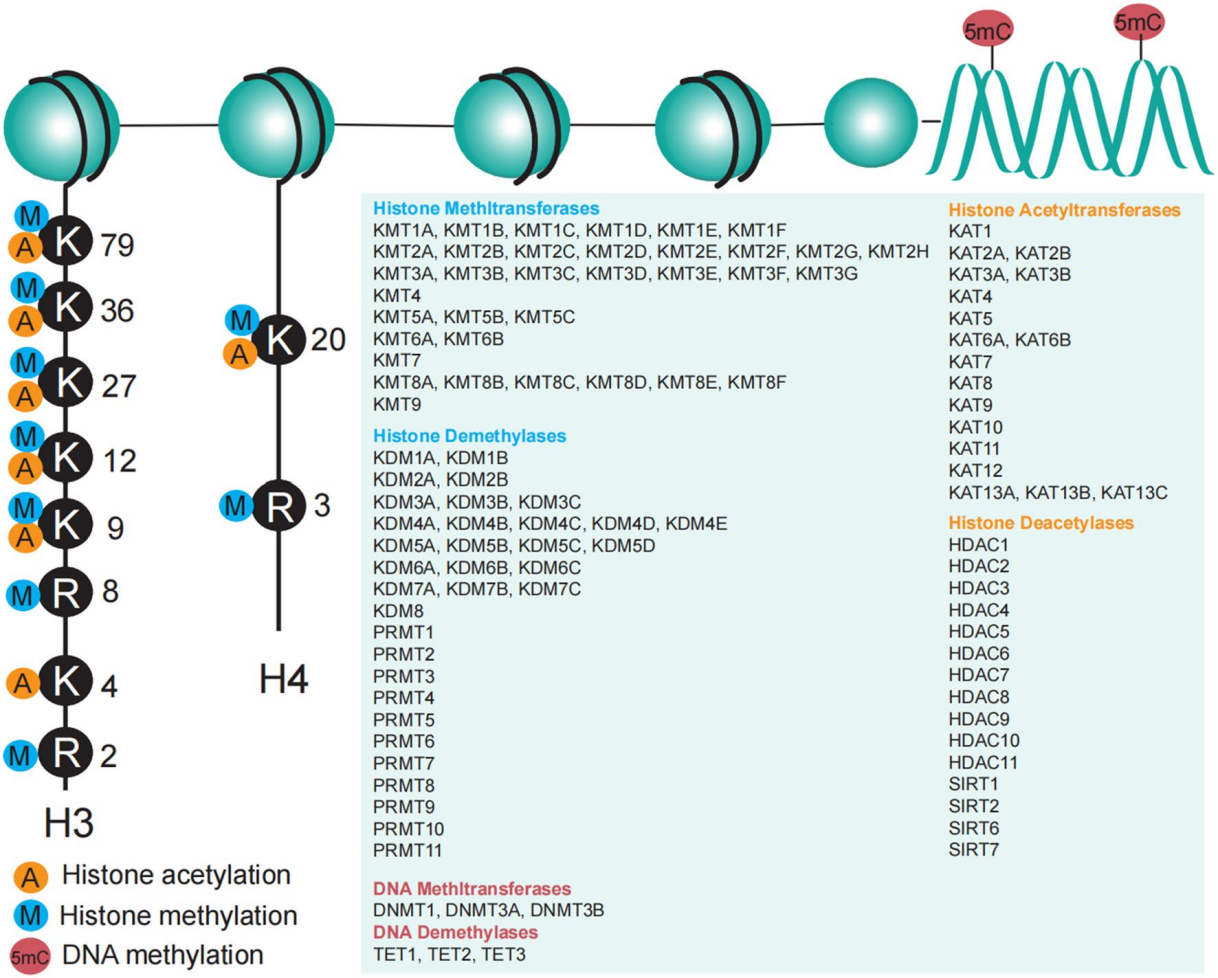
DNA/histone-modifying enzymes. DNA methylation and demethylation are dynamically regulated by DNA methyltransferases and demethylases. Histone methylation and demethylation are controlled by histone methyltransferases and demethylases. Histone acetyltransferases and deacetylases are responsible for histone acetylation and deacetylation, respectively

TFs and the cofactors are not only regulated by upstream transcriptional activation or repression but also controlled by post-translational modifications (PTMs), such as acetylation, phosphorylation, ubiquitination, SUMOylation [4]. Phosphorylation is the most common modification of TFs and the cofactors. Phosphorylation of TFs and the cofactors positively or negatively regulates its DNA accessibility, protein stability, and protein-protein interaction to influence transcriptional activity for expression of genes. Specific phosphorylated sites of TFs and the cofactors have different influences on their functions. TFs have been cataloged based on DNA-binding specificity, which has been classified into at least nine super classes, including basic domains, zinc-coordinating DNA-binding domains, helix-turn-helix domains, alpha-Helices exposed by beta-structures, other all-alpha-helical DNA-binding domains, immunoglobulin fold, beta-Hairpin exposed by an alpha/beta-scaffold, beta-Sheet binding to DNA, beta-Barrel DNA-binding domains, and other undefined DNA-binding domains [4]. In general, once the DNA binding domain is phosphorylated, it disturbs TFs to recognize specific DNA sequences and impairs transcriptional activity, while the phosphorylation of the regulatory domain in TFs is more complicated and need more evidence to determine it. More importantly, many TFs’ cofactors generally contain a regulatory domain with enzymatic activities, such as acetylation/deacetylation, methylation/demethylation, ubiquitination, and SUMOylation [5–8]. Mostly, these kinds of TFs’ cofactors modify histones via methylation and acetylation, except for DNA methylation, as it is summarized in Fig. 1. The enzymatic activities mainly function on genomic DNA and histone proteins and the interactors. With more and more enzymatic proteins found to act as transcriptional regulators or coregulators, the regulation of PTMs in these proteins, especially these phosphorylated sites in catalytic domains, significantly determines the transcriptional activity. However, the functions of phosphorylation on these DNA/histone-modifying enzymes are more complicated. In this review, we comprehensively describe how phosphorylation affects DNA/histone-modifying enzymes with specific sites, and focus on these phosphorylated sites which are directly associated with enzymatic activation or repression.

## Regulation of DNA methyltransferases by phosphorylation

In mammals, DNA methyltransferases are responsible for DNA methylation by adding a methyl group from S-adenosyl-methionine (AdoMet) to the fifth cytosine in CpG-enriched sequences of the genome. It consists of five members: DNMT1, DNMT3a, DNMT3b, DNMT2, and DNMT3L. Among them, DNMT1 is responsible for maintaining DNA methylation by converting hemi-methylated CpG dinucleotides in daughter strands to methylated status during the DNA replication process, while DNMT3A and DNMT3B are different from DNMT1. Both of them target unmethylated DNA strands and exert de novo methylation [9]. As for another two DNA methyltransferases, DNMT2 and DNMT3L, DNMT2 has residual DNA methyltransferase activity with preference of tRNA methylation [10, 11]. It is found that DNMT2 methylates cytosine 38 in the anticodon loop of tRNA16 [12]. DNMT3L is catalytically inactive, but it interacts with DNMT3A and DNMT3B, which stimulates their methylation activity [12].

It is crucial for DNA methyltransferases to epigenetically regulate genes via DNA methylation during the development of normal and cancer cells. Therefore, post-translational modifications of DNA methyltransferases modulate protein expression of these enzymes and the level of DNA methylation. Recent studies have evidenced that phosphorylation of DNA methyltransferases at N-terminal serine/threonine residues is likely to affect their enzymatic activity. DNMT1 is a multi-domain protein with a DMAP1-binding domain, RFTS domain, a CXXC domain, two BAH domains and a C-terminal catalytic domain. The N-terminal domains of DNMT1, DMAP1-binding domain and RFTS domain, are key for its stability and subcellular localization onto DNA replication sites [13, 14]. And also, enzymatic activity of DNMT1 is also regulated by RFTS domain and CXXC domain [15, 16]. UHRF1 interacts with DNMT1 through its SRA domain, which is essential for DNA methylation maintenance [17]. It has also been reported that UHRF1 not only facilitates DNMT1 to genomic loci by recognizing H3R2 and H3K9me2/3 mark, but also stimulates DNMT1 catalytic activity via UHRF1-dependent H3 ubiquitination [18, 19]. At the same time, DNMT1 is recruited by UHRF1 and forms a macromolecular complex with other proteins during the cell cycle, including PCNA, TIP60, HDAC1, SUV39H1, HAUSP, and pRB [20]. It is noticed that PCNA partly recruits DNMT1 to the replication sites [21]. Human DNMT1 is discovered to be phosphorylated at Ser 154 by cyclin-dependent kinases (CDKs) 1, 2, and 5, which is orthologous with mouse Dnmt1 Ser152 [22]. The mutation of DNMT1 at position 154 (S154A) severely impairs its methylation activity. However, it is still unknown whether phosphorylation of Ser154 promotes the interaction of the N- and catalytic domains of DNMT1 and thus increase DNMT1 activity for DNA hypermethylation. Another site, Ser515 of human DNMT1, has been shown to be phosphorylated during the cell cycle, which enhances DNMT1 methylation activity [23]. It is suggested that phosphorylation of Ser515 in DNMT1 is helpful for an interaction between N-terminus and catalytic domains of DNMT1. Furthermore, Ser127and Ser143 of DNMT1 have also been identified to be phosphorylated by AKT and PKC [24]. AKT-mediated phosphorylation of DNMT1 at Ser143 peaks during DNA synthesis and stabilizes DNMT1 from degradation [25]. The difference of these two kinases on DNMT1 is that the phosphorylation of DNMT1 at Ser127 by PKC disturbs the DNMT1/UHRF1 interaction without affecting the DNMT1/PCNA interaction, while AKT-mediated double phosphorylation of DNMT1 at Ser127 and Ser143, repress DNMT1/PCNA and DNMT1/UHRF1 interactions. Therefore, AKT/PKC-mediated phosphorylation of DNMT1 is considered to be a hallmark determining the interaction of DNMT1 with PCNA or UHRF1. In addition, mouse Dnmt1 is also reported to be phosphorylated at Ser146 by casein kinase 1δ/ε, which disrupts the DNA-binding activity of Dnmt1 but not alter its methylation activity and interaction of Dnmt1/PCNA [26]. The phosphorylation of human DNMT1 at the N-terminal nuclear localization signal (NLS) by protein kinase B (PKB) promotes its nuclear translocation in the condition of IL6 stimulation [27]. In summary, DNMT1 phosphorylation plays a central role in its methylation activity, protein stability, and the interaction with other proteins. Aberrant phosphorylation of DNMT1 results in fibroblast activation and an increase of α-smooth muscle actin and type I collagen [28]. Previous mass spectrometry results have identified several phosphorylation sites on DNMT1. Some of the phosphorylation sites have been functionally verified, but there are still unclear for the remaining of them. It is also interesting that phosphorylation sites of DNMT1 are dependent on cell status and cell types. For example, Ser154, Ser515, and Ser714 of DNMT1 are phosphorylated in HEK293T cells, Ser127, Ser143, and Ser714 of DNMT1 are found in Jurkat cells, and Ser143 of DNMT1 is in lung cancer cells [29–32]. DNMT1 is phosphorylated at Ser154 and Ser515 in the cell cycle, which significantly affects enzymatic activity and protein stability of DNMT1 [22, 23]. Glycogen Synthase Kinase 3 (GSK3) phosphorylates DNMT1 at Ser714 to block the methylation of unmethylated DNA [33].

The de novo DNA methyltransferase DNMT3a is also reported to be phosphorylated at two key residues (Ser386 and Ser389) by casein kinase 2 (CK2), which impairs the methylation ability of DNMT3a and switches DNMT3a to localize at heterochromatin [34]. The other site, Ser255 of DNMT3a, determines its intracellular localization to regulate erythrocytic differentiation [35]. It is shown that the extracellular signal-regulated kinase 1/2 (ERK1/2) phosphorylates DNMT3a at Ser255, resulting in DNMT3a translocate into the nucleus. As for DNMT3b phosphorylation, there are still no studies on it and it needs to be further explored.

## Regulation of DNA demethylases by phosphorylation

As for DNA demethylation, TET family proteins, including TET1, TET2, and TET3, can oxidize 5-methylcytosine (5mC) into 5-hydroxymethylcytosine (5hmC), 5-carboxylcytosine (5caC), and 5-formylcytosine (5fC) during the processes of DNA replication or thymine-DNA glycosylase (TDG) and base excision repair (BER) (TDG-BER) pathway [36–39]. TET proteins-mediated DNA demethylation involves in numerous biological processes, including stem cell differentiation, metabolism, and inflammation [40–42]. And TET1, TET2, and TET3 display distinct expression levels in different cell types and differentiation development [43]. TET1 and TET2 are mainly expressed in mouse stem cells for active DNA demethylation on gene’ promoters and bodies, while TET3 is enriched in the oocyte and neuronal tissues [44–46]. The activity of TET proteins is not only directly dependent on several molecules, such as Fe(II), 2-oxoglutarate, interactors, its inhibitors, and stimulators, but also influenced by various post-translational modifications (PTMs) [36, 47]. Among these PTMs, phosphorylation of TET proteins significantly impacts their enzymatic activity, protein stability and interactions, which is reported to be inhibited by glycosyltransferase OGT-induced O-GlcNAcylation [48].

Most phosphorylated sites of TET1 are located at its N-terminus by mass spectrometry, but the functions of these sites are still unclear [48]. Until now, phosphorylation of TET2 and TET3 is the most widely studied. TET2 is considered as a tumor suppressor for its effect on the DNA 5-hydroxymethylome [49]. Phosphorylation of TET2 at Ser99 by AMP-activated kinase (AMPK) enhances its tumor suppression by stabilizing TET2. High level of glucose impairs TET2 phosphorylation in diabetic patients, which suggests an epigenetic pathway by which a hyperglycemic environment induces cancers [49]. Homologous to murine Tet2 at Ser97, phosphorylation stabilizes Tet2 and promotes its interaction with 14-3-3β [50]. Tet2 phosphorylation at Ser97 or its mimicking mutant S97E can rescue differentiation defects in C2C12 cells by upregulating expressing of Pax7. In contrast, phosphorylation of TET2 at Y1902 by fibroblast growth factor receptor 3 (FGFR3) splicing mutant FGFR3_△7−9_, degrades TET2 via ubiquitination and promotes hepatocellular carcinogenesis [51]. TET2 is required for lineage commitment and differentiation of stem cells. It is reported that TET2 is phosphorylated by cytokine receptor-associated JAK2 at Tyr 1939 and 1964, which facilities stem cells to differentiate to erythroid cells by interacting with the erythroid transcription factor KLF1 [52]. The dioxygenase activity of TET3 is activated by its phosphorylation at Ser1310 and Ser1379 [53]. These two sites are highly conserved within their catalytic domain and phosphorylated by cyclin-dependent kinase 5 (CDK5). Interestingly, overexpression of TET3 phosphorylation mutant (S1310A/S1379A) leads to increased expression of metabolic genes, distinct from wild-type TET3 for neuron-specific genes. Therefore, phosphorylated and unphosphorylated TET3 display different binding affinities on histones for neuronal differentiation.

## The regulation of histone lysine and arginine methyltransferases by phosphorylation

**Fig. 2:**
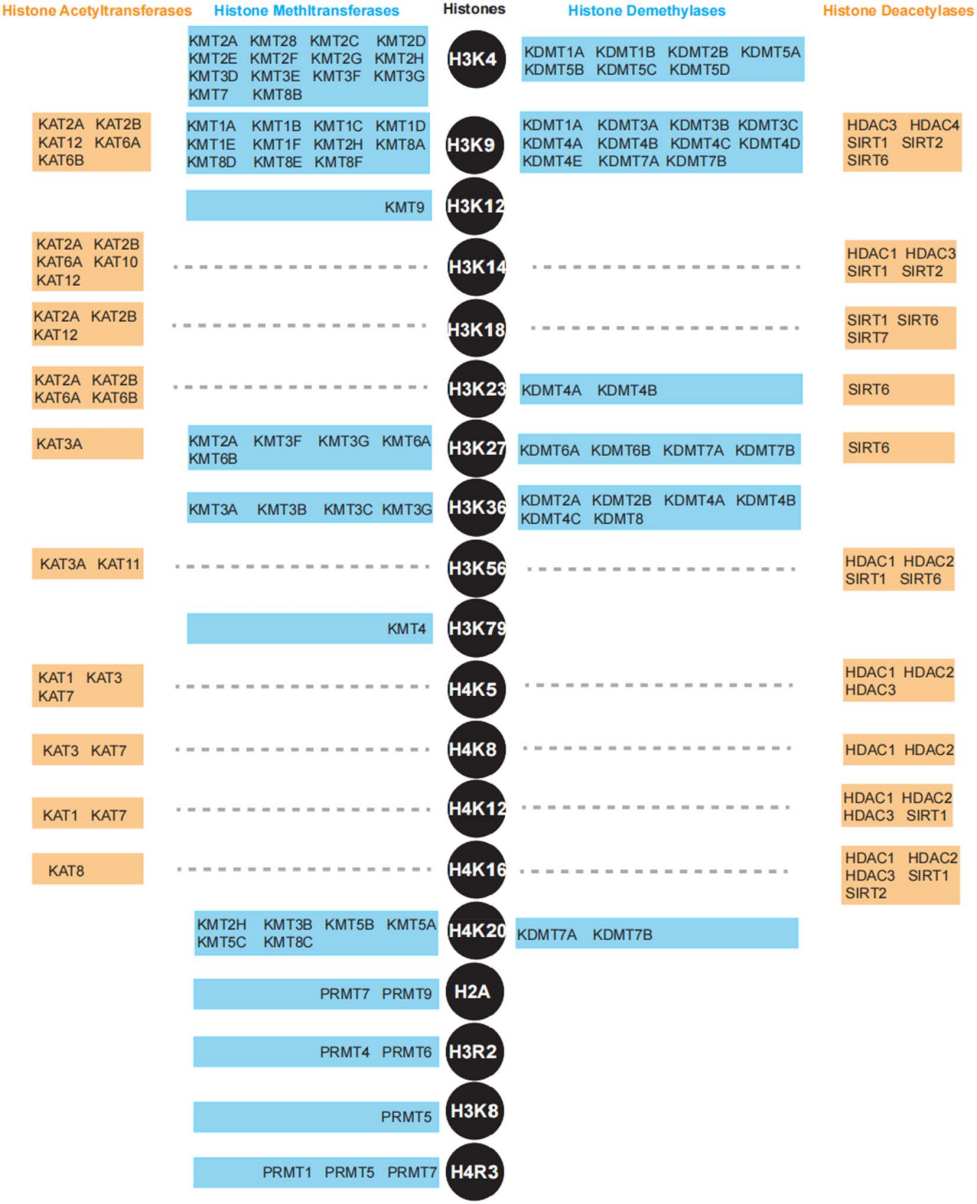
Different histone-modifying enzymes target different specific histone sites

Histone methylation commonly occurs by various lysine and arginine methyltransferases in a site-specific manner (Fig. 2) [54]. Lysine methyltransferases (KMTs) are responsible for mono-, di-, tri-methylated histone H3K4, H3K9, H3K27, H3K36, and H4K20, while arginine methyltransferases (PRMTs) are only for mono- or di-methylation on histone H3R2, H3R8, H4R3, and H2A [55, 56]. Specifically, several KMTs including SET1A, SET1B, ASH1, and MLL1-5, recognizes histone H3K4 for methylation; SUV39H1, SUV39H2, G9a, SETDB1, GLP, RIZ1, and CLL8 methylate histone H3K9; Histone H3K27 is the substrate of EZH2; SET2, SMYD2, and NSD1 exert their enzymatic activity on histone H3K36, and H3K36 can also be catalyzed by NSD2, SETMAR; DOT1L is for histone H3K79 methylation; SUV420H1, SUV420H2, SET7, and SET8 methylate H4K20, as shown in Table 1. The methylation activity of histone lysine and arginine methyltransferases is associated with human diseases, including prostate, breast, lung cancers and the responses to environment stress [57–59]. Specifically, histone methylation can regulate the tightness of the nucleosome in most case, and thus affect the access of transcription factors and RNA polymerase to their targeted genes [60]. Phosphorylation of catalytic domain of KMTs is one of the main factors to generally suppress or activate their methyltransferase activity on histones. However, only a few of KMTs have been reported to be phosphorylated (Table 1).

KMT1A, known as SUV39H1, is phosphorylated at residues Tyr297 (mouse Tyr303 and flies Tyr308) by receptor-type tyrosine kinase ERBB4, which enhances the tri-methylation activity of histone H3K9 [61]. And the protein level of SUV39H1 peaks at the S phase and maintains from S to M phases in the cell cycle, in which SUV39H1 is phosphorylated by CDK2 at Ser391. The phosphorylation of SUV39H1 at Ser391 aims to dissociate from chromatin and prepares for histone demethylase JMJD2A occupancy [62]. KMT1C (G9a) is phosphorylated at Ser591 by ATM and colocalizes with GLP1 (also known as EHMT1) under the circumstance of DNA double-strand breaks (DSBs) [63]. The methylation activity of G9a is essential for the DNA repair pathway. It suggests that inhibition of G9a enzymatic activity by phosphorylation improves the DNA mutation rate and effects from DNA breaks. Other studies also show that G9a is phosphorylated at Ser 211 by CK2 and then directly interacts with replication protein A (RPA) on chromatins [64]. By forming the complex of RPA and G9a, it facilitates homologous recombination (HR) for DNA repair. The human mixed-lineage leukemia 1 (MLL1) is reported to be phosphorylated at Ser136 and Ser142 by CK2, which significantly enhances its interaction with a transcription coactivator LEDGF/p75 [65]. Interaction of LEDGF/p75 and MLL1 not only impacts HIV integrase for active transcription of viral genes, but also regulates acute leukemia development via MLL1 translocation fusion. It is also reported that T2724 and S2726 at taspase1-dependent cleavage domain of full MLL1 are phosphorylated by CK2, which blocks taspase1-dependent cleavage of MLL1 and results in the destabilization of MLL1 [66]. MLL1 stability is important for aggressive leukemia and phosphorylation-mediated degradation of MLL1 is likely to be a potential treatment for acute leukemia. As for Leukemogenic MLL, it takes advantage of the C-terminal SET domain to methylate histone H3 lysine 4 (H3K4), while its N-terminal domain is composed by fusing more than 60 partners and processed to generate heterodimerized MLL [67, 68]. It is verified to be phosphorylated at Ser561 by ATR in the S phase of the cell cycle and therefore promotes its dissociation from chromatin and degradation by SKP2 E3 ligase in response to genotoxic stress [69]. MLL2 (KMT2B) is phosphorylated at Thr542 by CDK2 in the late G1 phase of the cell cycle [69]. It attributes to MLL2 activation on H3K4me3 and promotes pluripotent stem cells differentiation. There are no reported phosphorylated sites of MLL3. MLL4 is reported to be phosphorylated at Ser1331 by AKT, which inhibits its methylation activity [70]. The enzymatic activity of MLL4 can be stimulated by PI3Kα inhibitors and thus it elicits a robust compensatory increase in ER-dependent transcription that limits therapeutic efficacy in ER-positive breast cancer. MLL5 is also phosphorylated and C2 targets Thr912 of MLL5 at the cell cycle [71]. It specifically occurs at the G2/M phase for entrance into mitosis through dissociation from condensed chromosomes, suggesting that subcellular localization of MLL5 is dependent on CDC2 activity. SMYD2 is reported to interact with CDK4/6 and is phosphorylated for methylation activation of H3K4 and H3K36. However, it is still not clear which sites of SMYD2 are phosphorylated. It is shown that histone dimethyltransferase WHSC1 is phosphorylated at Ser172 by activated AKT in prostate cancer, which prevents WHSC1 from degradation and subsequently activates transcription of RAC1 to drive cancer metastasis [72]. Phosphorylation of DOT1L by CDK1 at Ser1105 significantly impacts its subcellular localization and enzymatic activity In ES cells [73]. Specifically, phosphorylated DOT1L on this site cannot be present in the nucleus compared with that of wild type. And it also impairs DOT1L methylation activity so that the low methylation level of H3K79 fails to differentiate ES cells. Polycomb group proteins (PcG) PRC2-Ezh1α/β signaling pathway plays a crucial role in maintaining cell memory via H3K37me3 [74]. It is reported that Ser560, localized at the C-terminus of mouse zeste homolog 1β (Ezh1β), is phosphorylated in the cytoplasm and it promotes degradation of EZH1β by ubiquitin E3 ligase NEDD4 in the condition of oxidative stress [75]. CDK4/6-mediated phosphorylation of EZH2 at Thr345 enhances STAT3 methylation in keratinocytes, which activates STAT3 to induce the expression of a key proinflammatory transcription factor, IκBζ in psoriasis [76]. It is reported that EZH2, also called KMT6A, is phosphorylated at serine 21 by AKT, which abrogates histone H3 and reduces H3K27 trimethylation [77]. The phosphorylation of EZH2 at Ser21 functionally promotes cell growth and oncogenesis. In addition, there are still many sites identified to be phosphorylated in EZH2 and different phosphorylation sites function diversely in Table 1.

Protein arginine methyltransferases 1 (PRMTs) are a kind of enzymes, which are mainly responsible for histone arginine methylation in cells and regulate the cell cycle and cell proliferation by remodeling chromatin status. Phosphorylation of PRMTs is essential for its enzymatic activity and protein stability. It is reported that PRMT1 is phosphorylated at Ser297 in normal liver cells, while it is dephosphorylated by PP2A at this site in response to alcohol and other oxidative stresses [78]. It suggests that PRMT1 plays an important role in protecting liver patients from alcoholic disease. But Tyr291 phosphorylation inhibits PRMT1’s ability to methylate histone proteins and interacts with heterogeneous nuclear ribonucleoproteins (hnRNP A1 and hnRNP H3) in K562 cells [79]. PRMT6 is reported to be phosphorylated at Ser11 and Thr21 by CK2, which protects it from degradation and promotes RCC1 arginine methylation for tumorigenicity of glioblastoma stem cells [79].

**Table 1:** Phosphorylation of histone methyltransferases and demethylases, and its effect on the enzymatic activity

Isoforms of KMTs	Synonyms	Target residues	Kinases	Functional regulation upon phosphorylation	Involved in diseases and signaling pathways	References
KMT1A	SUV39H1	Tyr297	ERBB4	Activation of enzymatic activity; Chromatin remodeling	NRG-1/ErbB4 signaling	[61]
		Ser391	CDK2			
KMT1C	G9a/ EHMT2	Ser211	CK2	Suppression of its binding affinity for RPA70	DNA damage repair	[63]
		Ser591	ATM	Inhibition of enzymatic activity; Suppression of the recruitment of DNA repair factors	DNA damage repair	[80]
KMT2A	MLL1	Ser136	CK2	Increase of the affinity of IBMs for the LEDGF/p75 IBD	MLL-rearranged leukemia	[64]
		Ser142				
		Thr2724	CK2	Degradation	MLL-rearranged leukemia	[65]
		Ser2726				
KMT2B	MLL2	Thr542	CDK2	Chromatin remodeling	Cell cycle	[69]
KMT2D	MLL4	Ser1331	AKT	Inhibition of enzymatic activity	PI3K pathway	[59]
KMT2E	MLL5	Thr912	CDC2	Activation of enzymatic activity	Cell cycle	[81]
KMT4	DOT1L	Ser1105(Mouse)	CDK1	Inhibition of enzymatic activity	Embryonic stem cell (ESC) differentiation	[73]
KMT6A	EZH2	Ser21	AKT	Inhibition of enzymatic activity	Oncogenesis	[77]
		Ser220	MELK	Degradation	Natural killer/T-cell lymphoma (NKTL)	[82]
		Tyr244	JAK3	Promotion of the dissociation of the PRC2 complex	Natural killer/T-cell lymphoma (NKTL)	[83]
		Thr261	CDK5	Degradation	Pancreatic cancer cell migration and invasion	[84]
		Thr311	AMPK	Disruption of the interaction with SUZ12	Tumor growth	[85]
		Thr350	CDK4/6	Activation of enzymatic activity	Psoriasis	[76]
		S363	GSK3β	Inhibition of enzymatic activity	Human breast cancers	[86]
		T416	CDK2	Chromatin remodeling	Triple-negative breast cancer; Cell cycle	[87]
		T492	CDK1	Degradation	Lung cancer cell migration and invasion	[88]
		Y646	JAK2	Degradation	Lymphoma pathogenesis	[89]
KMT6B	EZH1	Ser560(Mouse)	Unknown	Degradation	Adaptive stress response pathway	[75]
PRMT1		Tyr291	Unknown	Inhibition of enzymatic activity	Mitosis; Tumorigenicity; Radiation response of glioblastoma stem cells	[79]
		Ser297	Unknown	Activation of enzymatic activity	Inflammation	[78]
PRMT6		Ser11	CK2	Stabilization	Mitosis; Tumorigenicity; Radiation response of glioblastoma stem cells	[79]
		Thr21				
KDM2A	CXXC8 FBL7 FBXL11 JHDM1A KIAA1004	Thr632	ATM	Disruption of its binding to chromatin	DNA damage repair	[90]
KDM2B	CXXC2 FBL10 FBLX10 JHDM1B PCCX2	Ser265	PKA	Chromatin remodeling	Cell cycle	[91]
KDM4B	JMJD2B JHDM3B	Ser666	PKA	Enhance of its binding to SF3B3	Androgen deprivation	[92]
		Thr305	ERK	Stabilization	Colon cancer	[93]
		Ser352				
		Ser566				
		Thr1065				
KDM4C	JMJD2C JHDM3C	Ser918	PKR	Degradation	Glioblastoma Tumorigenesis; Wnt/β-catenin signaling	[94]
KDM5B	JARID1B	Ser1456	CDK1	Disruption of its binding to chromatin	Triple negative breast cancer	[95]

## The regulation of histone lysine and arginine demethylases by phosphorylation

Histone lysine demethylases (KDMs), in contrast to KMTs, function to remove methyl groups of methylated histone proteins. The alters on histone methylation by KDMs regulate gene expression in the processes of development and tumor growth [96]. And KDMs can be considered as tumor oncogene or tumor suppressor, which is determined by KDMs-targeted genes. KDMs are composed of eight superfamilies (KDM1-8) and targets various methylated histone proteins. KDM2A contains a JmjC domain at its N-terminus and a PHD zinc finger, a CxxC-type zinc finger at C-terminus. Thr632 at the PHD zinc finger of KDM2A is phosphorylated by ATM kinase, which impairs its chromatin-binding capacity [90]. KDM3A also undergoes phosphorylation at Ser265 by protein kinase A (PKA) and transcriptionally regulates cell-cycle genes in response to DNA damage [91]. KDM4B is phosphorylated by PKA at Ser666 and it promotes castration-resistance by weakening its binding to a splicing factor SF3B3 [92]. Protein kinase R phosphorylates KDM4C at Ser918, resulting in KDM4C ubiquitination and degradation [94]. But WNT3a can interrupt phosphorylation of KDM4C via GSK3-dependent protein kinase R inactivation. Phosphorylation of KDM5B at Ser1456 by cyclin-dependent kinase 1 (CDK1) abolishes its binding to the promoters of several pluripotency genes [95]. It is reported that the phosphorylation of KDM8 is decreased in KDM4C knockdown cells, but the specific phosphorylated sites are unknown [97]. Until now, it remains controversial about the existence of arginine demethylases [98]. Recently, several lysine demethylases, including KDM3A, KDM4E, KDM5C, have been reported to demethylate methylarginine in vitro [99]. However, there are no phosphorylated sites reported on these three enzymes. In summary, the effects of phosphorylation on these demethylases are not enough. Further investigation is necessary to explore the function of phosphorylation on these lysine/arginine demethylases.

## The regulation of histone acetyltransferases by phosphorylation

Histone acetyltransferases (HATs) are major players in epigenetically modulating gene transcription via acetylation on histones (Fig. 2). They recognize and transfer an acetyl group from acetyl CoA to acetylate lysine residues of histones, which generally occurs at gene promoters. And substrates of HATs are not only histones, but also other non-histone proteins, such as p53, GATA1, and erythroid Kruppel-like factor (EKLF) [100–102]. Abnormal HATs expression directly or indirectly leads to many human diseases, such as cancers and neurodegenerative disease. Phosphorylation of HATs plays a pivotal role in its enzymatic activity and protein stability (Table 2). HAT1, also called KAT1, is reported to be phosphorylated by active adenosine monophosphate (AMP)-activated protein kinase (AMPK) at Ser190, which enhances its acetylation activity on histone H4 and inhibits DNMT1 binding to relaxed chromatin [103]. GCN5 (KAT2A) plays a dual role in the deeding-to fasting transition and is phosphorylated by PKA at Ser275 [104]. In the fasted condition, GCN5 induces gluconeogenesis and acetylates PGC1α to repress the activity of PGC1α. In order to switch to the fed state, GCN5 is phosphorylated by PKA in the complex of GCN5-CITED2-PKA and then turn to acetylate histone H3.

Until now, P300 is the most studied protein about its functions influenced by phosphorylation. Among these phosphorylated sites we have summarized, phosphorylation of Ser1834 by AKT, Ser2271, Ser2279, Ser2291, and Ser2315 by mTOR, and Ser2279, Ser2315, and Ser2366 by extracellular signal-regulated kinase 2 (ERK2), significantly enhances P300 acetylation activity [105–107]. P300 phosphorylation by AKT at Ser1834 enhances its acetylation activity on its adaptor factor, alteration/deficiency in activation 3 (ADA3), and it involves in growth factor-associated cell cycle by epidermal growth factor receptor (EGFR) activation [105]. MTOR-mediated phosphorylation of P300 at its C-terminal domain prevents the catalytic domain from binding to the RING domain, which reduces starvation-induced cell autophagy and lipogenesis [108]. P300 phosphorylation by ERK2 at the C terminus is essential for its recruitment to the promoter region of keratin 16 and cooperatively interacts with SP1 and c-Jun for upregulation of keratin 16 [109]. And also, ERK2-induced P300 phosphorylation stimulates acetylation of the nuclear factor of activated T-cells c1 (NFATc1) and activation of the myosin heavy chain 1 (MYHC1) expression during the transformation of skeletal muscle fiber type [110]. Other phosphorylated sites, such as Ser106 by ataxia-telangiectasia mutate (ATM) involved in DNA damage, Ser1038/2039 by CDK1 stabilizing P300, and Ser89 by CK2 or SIK2 influencing downstream gene transcription [111–113]. In addition, the HAT activity of P300 is also dynamic in the differentiation of mouse F9 embryonal carcinoma cells. It is reported that the HAT activity of P300 is only dependent on P300 phosphorylation in differentiated F9 cells, although P300 is also strongly phosphorylated in undifferentiated F9 cells [114].

AKT also phosphorylates the acetyltransferase p300/CBP-associated factor (PCAF) and increases its acetylation on high-mobility group AT-hook 2 (HMGA2) at lysine 26 (K26) for esophageal squamous cell carcinoma growth [106]. The phosphorylation of CBP at Ser436 by a typical protein kinase C (aPKC), homologous to P300 phosphorylation G422S functions to increase new neurons’ survival and thus promotes hippocampal neurogenesis [107]. It is explained that maybe p300 phosphorylation weakens its interaction with CREB. Insulin also suppresses the formation of the complex of CREB-CBP by phosphorylating CBP, resulting in aberrant hepatic glucose production (HGP) [115]. And it is confirmed again that p300 prefers binding to unphosphorylated CBP. As for TIP60 phosphorylation, only two sites, Ser86 and Ser90, have been reported. Phosphorylation of TIP60 at Ser86 by GSK3 promotes its enzymatic activity on histone H4 and P53 acetylation, resulting in apoptosis [116]. While phosphorylation at Ser90 of TIP60 by CDK9 enhances its binding to chromatin [117]. It is also found that TIP60 phosphorylation at Ser90 by cyclin B/CDC2 can arrest cells at the G2/M phase of the cell cycle [118]. Other KATs, including KAT7 and KAT13A, are also reported to be phosphorylated. Specifically, KAT7 (HBO1) is phosphorylated at Ser57 by polo-like kinase 1 (PIK1) to drive the transition of the cell cycle from G1 to S phase, and then HBO1 is phosphorylated by CDK1 at Thr85 and Thr88 during mitosis [116]. Significantly, it is evidenced that HBO1 phosphorylation by CDK1 provides a docking site for PIK1 binding. The protein stability of KAT7 is also regulated by phosphorylation. Protein kinase D1 (PDK1) can directly phosphorylate KAT7 at Thr97 and prevent KAT7 from ubiquitination-mediated degradation [119]. The predicted phosphorylation sites of KAT13A are numerous, but we have only summarized experimentally-verified sites in Table 2, as well as other KATs proteins.

**Table 2:** Phosphorylation of histone acetyltransferases and its effect on the enzymatic activity

Isoforms of KMTs	Synonyms	Target residues	Kinases	Functional regulation upon phosphorylation	Involved in diseases and signaling pathways	References
KAT1	HAT1	Ser190	AMPK	Activation of enzymatic activity	Mitochondrial biogenesis and function	[112]
KAT2A	GCN5	Ser275	PKA	Activation of enzymatic activity	Hepatic glucose metabolism	[104]
KAT2B	PCAF	Unknown	AKT	Activation of enzymatic activity	Esophageal squamous cell carcinoma	[106]
KAT3A	CBP	Ser436	PKC	Disruption of its binding to chromatin	Hippocampal neurogenesis	[94]
KAT3B	P300	Ser12	MAPK	Activation of enzymatic activity	Cancer-Induced Muscle Wasting	[118]
		Ser89	AMPK	Inhibition of enzymatic activity	Endothelial cell inflammation	[120]
			PKC	Disruption of its binding to chromatin	Cell growth and differentiation	[111]
			SIK2	Disruption of its interaction with PPARα	Hepatic lipid homeostasis	[112]
		Ser106	ATM	Stabilization of NBS1	DNA damage	[110]
		Ser1038	CDK1	Degradation	Lung cancer	[113]
		Ser2039				
		Ser1834	AKT	Activation of enzymatic activity	Cell cycle	[105]
		Ser1849	Unknown	Activation of enzymatic activity		[121]
		Thr1851				
		Thr1854				
		Thr1857				
		Thr2279				
		Ser2271	mTOR	Activation of enzymatic activity	Autophagy and Lipogenesis	[108]
		Ser2279				
		Ser2291				
		Ser2315				
		Ser2279	ERK2	Activation of enzymatic activity; Enhance of its interaction with SP1	Signal-regulated kinase 1 and 2 (ERK1/2) signaling	[109]
		Ser2315				
		Ser2366				
KAT5	TIP60	Ser86	GSK3	Activation of enzymatic activity	Apoptosis; PI3K signaling	[122]
		Ser90	CDK9	Enhance of its binding to chromatin	Cell proliferation	[117]
			Cyclin B/CDC2	Activation of enzymatic activity	Cell cycle	[123]
KAT7	HBO1	Ser50	ATR	Enhance of its binding to chromatin	Nucleotide excision repair	[124]
		Ser53				
		Ser57	PIK1	Enhance of its binding to chromatin	Cell cycle	[116]
		Ser85	CDK1			
		Ser88	CDK1			
			Cyclin E/CDK2	Enhance of its binding to chromatin	Cell cycle	[125]
		Thr97	PKD1	Stabilization	Cell proliferation	[119]
		Thr331				
KAT8	MOF	Thr392	ATM	Disruption of its interaction with 53BP1	Double-strand break repair	[126]
KAT13A	SRC1	Thr426	CDKs	Enhance of its interaction with PR	Breast cancer	[127]
		Ser22	Cyclin A2/CDK2			
		Ser395				
		Ser569				
		Ser1033				
		Thr1179				
		Thr1185				
		Thr1426	CDK1/2			

## The regulation of histone deacetylases by phosphorylation

Histone acetylation and deacetylation are dynamic processes dependent on cell states. Histone acetylation and deacetylation are performed by KAT family proteins and histone deacetylases (HDACs). There are four classes of HDACs based on their homologous protein sequences: the class I RPD3-like proteins including HDAC1, HDAC2, HDAC3, and HDAC8; class II HDA1-like proteins are HDAC4, HDAC5, HDAC6, HDAC7, HDAC9, and HDAC10; class III sirt2-like proteins containing SIRT1, SIRT2, SIRT3, SIRT4, SIRT5, SIRT6, and SIRT7; class IV protein is HDAC11. Many HDACs, which belong to class I, II, IV, require a zinc ion to deacetylate acetylated lysine, but for the class III HDACs, the proteins require NAD^+^ as a cofactor for the enzyme activity. In addition, other transcription factors, such as TCF1 and LEF1, is discovered recently to own the ability of deacetylating H3K9ac and H3K27ac [128]. But here, we do not discuss these two proteins without known phosphorylation sites.

In class I HDACs, HDAC1 is phosphorylated by various protein kinases and the specific sites directly influence HDAC1 subcellular localization, enzymatic activity and protein stability (Table 3). One of the most studied sites is Ser421 of HDAC1. It is reported to be targeted by different protein kinases. When it is phosphorylated by CK2, along with phosphorylation of Ser423, enhances its deacetylation activity on histones and also leads to nuclear accumulation of HDAC1 in response to neurotoxic [129, 130]. Other studies have found that Ser421 of HDAC1 can be phosphorylated by nemo-like kinase (NLK), which negatively regulates the WNT signaling pathway by inactivation of β-Catenin/LEF1 transcription [131, 132]. In the zebrafish central nervous system, HDAC1 is phosphorylated at Ser406 by Aurora A/B kinase, which affects its enzymatic activity and occurs during mitosis [133]. Among other sites, only Tyr72 is discovered to be phosphorylated by EGFR, and its phosphorylation maintains HDAC1 stability and its anti-apoptosis in tumors [134]. Different from HDAC1 phosphorylation at Ser421 on its enzymatic activity, Ser394, Ser422, and Ser424 of HDAC2 can be phosphorylated by CK2 and inhibits its deacetylation activity [135–137]. Furthermore, P21 is a key factor for vascular remodeling and its expression is regulated by a complex of HDAC2, retinoic acid receptor (RAR), and kruppel-like factor 5 (KIF5). Phosphorylation of HDAC2 dissociates it from RAR and deacetylates KIF5, resulting in transcriptional activation of P21 [137]. Single phosphorylation of HDAC2 at Ser394 enhances its interaction with HSP70 and fails to dephosphorylate HDAC2 by weakening the binding of PP2CA [138]. For HDAC3 phosphorylation, it is interesting that phosphorylated Ser424 increases HDAC3 activity, but several protein kinases are reported to be involved in, including CK2, TANK-binding kinase (TBK1), leucine-rich repeat kinase 2 (LRRK2), c-Jun N-terminal kinase (JNK), and PTEN-induced putative kinase 1 (PINK1) [139–143]. The other site, Ser374, is phosphorylated by homeodomain-interacting protein kinase 2 (HIPK2), which inhibits the enzymatic activity of HDAC3 [144]. Similarly, the enzymatic activity of HDAC8 is repressed by PKA-mediated phosphorylation at Ser39 [145, 146]. In addition, Ser39, Ser43, and Ser63 of HDAC8 are shown to be phosphorylated by activated AMPK under the condition of glucose deprivation in cancer cells, connecting cancer survival with glycogen pathway via overexpression of phosphoglucomutase 1 (PGM1) [147].

Phosphorylation by various serine/threonine kinases alters the subcellular localization of HDACs. HDAC4, as one of class II HDACs, is commonly verified to be phosphorylated at Ser246, Ser467, and Ser632 by calmodulin kinase II (CaMKII), which lead to nuclear export of HADC4 to the cytoplasm [148–150]. Another site, Ser740 of HDAC4, can be phosphorylated to prevent HDAC4 from degradation and it is phosphorylated by PKA [151]. It has been known that 14-3-3 binding sites of HDACs is crucial for nuclear-cytoplasmic shutting. Phosphorylation of HDAC5 at Ser259 and Ser498 by protein kinase D (PKD), as well as Ser218 and Ser448 of HDAC9, contributes to the formation of 14-3-3 binding sites. But it is only verified at cardiomyocytes and myocyte-like cells [152, 153]. The two sites of HDAC5, Ser259 and Ser498, are also reported by other kinases, including AMPK, AKT, CaMKII in cells with different types [154–157]. Although kinases-phosphorylated HDACs at specific sites partly promote the binding of 14-3-3 to HDACs and stabilize HDACs, the subcellular localization of HDACs is not strictly controlled by phosphorylation. Different from other HDACs, Ser178-phosphorylated HDAC7 exists in both the nucleus and the cytoplasm, while Ser344 and Ser479-phosphorylated HDAC7 is only localized in the nucleus [157]. The enzymatic activity of HDAC6 is reported to be significantly affected by phosphorylation in previous studies. Different kinases-mediated phosphorylation of HDAC6 at Ser21, Ser22, Ser458, Ser1035 activates its ability to deacetylate histones [158–161]. Only Tyr570 of HDAC6 is phosphorylated to inhibit its deacetylation activity [162].

**Table 3:** Phosphorylation of histone deacetylases (HDAC1-11) and its effect on the enzymatic activity

Isoforms of KMTs	Target residues	Kinases	Functional regulation upon phosphorylation	Involved in diseases and signaling pathways	References
HDAC1	Thr65	CaMKII	Activation of enzymatic activity	Heart failure	[163]
	Ser69				
	Ser85				
	Thr195				
	Ser197				
	Thr355				
	Tyr72	EGFR	Stabilization	Cancer cell survival	[134]
	Ser406	Aurora A/B	Activation of enzymatic activity	Zebrafish embryos development	[133]
	Ser423	CK2	Enhance of its interaction with SIN3A	Cell cycle	[164]
	Ser421	CK2			
		NLK	Disruption of its interaction with LEF1	WNT signaling pathway	[131]
HDAC2	Ser407	CK2	Disruption of its interaction with RARα	Proliferation of vascular smooth muscle cells	[137]
	Ser394	CK2	Inhibition of enzymatic activity	Inflammation	[136]
	Ser422				
	Ser424				
HDAC3	Ser374	CaMKII	Inhibition of enzymatic activity	Heart failure	[144, 163]
		HIPK2	Inhibition of enzymatic activity	Colorectal carcinoma and sepsis	[144]
	Ser424	CK2	Activation of enzymatic activity	Tumorigenesis	[139]
		TBK1	Activation of enzymatic activity	Innate antiviral immunity	[140]
		LRRK2	Activation of enzymatic activity	Parkinson’s disease	[140]
		c-Jun N-terminal kinase	Activation of enzymatic activity	Triple-negative breast cancer	[142]
		PINK1	Activation of enzymatic activity	Parkinson’s disease	[143]
HDAC4	Ser246	TBK1/IKKε	Suppression of IRF3 phosphorylation	Innate immune	[165]
		CaMKIV	Enhance of its interaction with 14-3-3	DNA binding	[166]
		PKD	Nuclear extrusion into the cytoplasm	Mitogenic signaling	[167]
		CaMKII	Disruption of its interaction with MEF2	Skeletal muscle hypertrophy	[148]
	Ser467	CaMKIV	Enhance of its interaction with 14-3-3	DNA binding	[166]
		CaMKII	Nuclear extrusion into the cytoplasm	Metabolism	[149]
	Ser632	CaMKIV	Enhance of its interaction with 14-3-3	DNA binding	[166]
		PKD	Nuclear extrusion into the cytoplasm	Mitogenic signaling	[167]
		PP2A	Nuclear extrusion into the cytoplasm	Neurodegeneration	[168]
	Ser740	PKA	Stabilization	Bone and chondrocyte development	[151]
HDAC5	Ser259	AMPK	Nuclear extrusion into the cytoplasm	WNT signaling pathway	[155]
		PKD	Enhance of its interaction with 14-3-3	Cardiac myocyte hypertrophy	[153]
		AKT	Nuclear extrusion into the cytoplasm	Vascular disorders	[155]
		CaMKII	Nuclear extrusion into the cytoplasm	Diabetes	[156]
		SIK1	Chromatin remodeling	Cardiac hypertrophy	[169]
		PKCδ	Nuclear extrusion into the cytoplasm	Heart failure	[170]
	Ser280	PKA	Disruption of its interaction with 14-3-3	cAMP/PKA signaling pathway	[171]
	Thr292	PRK1	Disruption of its interaction with 14-3-3		[172]
	Ser493	GRK5	Nuclear extrusion into the cytoplasm	Heart failure; NF-κB signaling pathway	[173]
	Ser498	AMPK	Nuclear extrusion into the cytoplasm	WNT signaling pathway	[154]
		PKD	Enhance of its interaction with 14-3-3	Cardiac myocyte hypertrophy	[153]
		AKT	Nuclear extrusion into the cytoplasm	Vascular disorders-	[155]
		PKA	Nuclear extrusion into the cytoplasm	Heart failure	[174]
	Tyr642	FAK	Nuclear extrusion into the cytoplasm	Osteocyte mechanotransduction	[174]
	Ser661	PKA	Nuclear extrusion into the cytoplasm	Heart failure	[170]
	Ser611	Unknown	Enhance of its interaction with other proteins	Cell cycle	[175]
	Ser755				
	Ser1108				
HDAC6	Ser21	GRK5	Activation of enzymatic activity	Cancer	[158]
	Ser22	GSK3β	Activation of enzymatic activity	Parkinson’s disease	[159]
	Ser289	ASK1	Stabilization	Retinopathy of prematurity	[176]
	Thr293				
	Ser458	CK2	Activation of enzymatic activity	Aggregation of misfolded proteins	[160]
	Tyr570	EGFR	Inhibition of enzymatic activity	Receptor endocytosis and degradation	[162]
	Ser1031	ERK1		Cell migration	[161]
	Ser1035	ERK1	Activation of enzymatic activity		
HDAC7	Ser155	PKD1	Nuclear extrusion into the cytoplasm	Apoptosis	[177]
	Ser178	PKD1	Nuclear extrusion into the cytoplasm	Cell proliferation and migration; VEGF signaling pathway	[178]
		CaMKI	Nuclear extrusion into the cytoplasm	Angiogenesis	[179]
		CRM1	Nuclear extrusion into the cytoplasm	Myogenesis	[180]
	Ser181	PKD1	Nuclear extrusion into the cytoplasm	Bone formation and maintenance	[181]
	Ser318	PKD1	Nuclear extrusion into the cytoplasm	Apoptosis	[180]
	Ser344	PKD1	Nuclear extrusion into the cytoplasm	Cell proliferation and migration; VEGF signaling pathway	[177]
		CaMK I	Nuclear extrusion into the cytoplasm	Angiogenesis	[178]
	Ser448	PKD1	Nuclear extrusion into the cytoplasm	Apoptosis	[180]
	Ser479	PKD1	Nuclear extrusion into the cytoplasm	Cell proliferation and migration; VEGF signaling pathway	[181]
		CaMKI	Nuclear extrusion into the cytoplasm	Angiogenesis	[177]
HDAC8	Ser39	AMPK	Nuclear extrusion into the cytoplasm	Lung cancer cell survival	[147]
		PKA	Inhibition of enzymatic activity		[182]
	Ser43	AMPK	Nuclear extrusion into the cytoplasm	Lung cancer cell survival	[147]
	Ser63	AMPK			
HDAC9	Ser218	PKD	Disruption of its interaction with 14-3-3	Cardiac hypertrophy	[152]
	Ser448				

SIRT (1–7) belongs to the class III HDACs with nicotine adenine dinucleotide (NAD^+^) as a cofactor. It is originally discovered in yeast and characterized to deacetylate histones for transcription repression [183]. Structurally, all SIRT proteins share a highly conserved catalytic domain which forms a clef for the substrates and nicotinamide. Due to that SIRT3, SIRT4 and SIRT5 localize in mitochondria, the roles of these phosphorylated proteins on histone are not clarified. Therefore, only SIRT1, SIRT2, SIRT6, and SIRT7 are discussed (Table 4). Phosphorylated SIRT1 is involved in multiple cellular processes, including metabolism, DNA repair, apoptosis, inflammation, and aging. It is reported that mammalian sterile 20-like kinase 1 (MST1) overexpression leads to DNA damage-induced apoptosis by upregulating P53 acetylation [184]. P53 acetylation accounts for inhibiting the deacetylation of SIRT1 by MST1-mediated phosphorylation in the C-terminus of SIRT1. But SIRT1 phosphorylation at Ser47 by mTOR can rescue cells from DNA damage-induced senescent by elevating p65/RelA NF-κB acetylation [185]. The phosphorylation of two sites of SIRT1, Ser47and Thr522, have been reported to promote SIRT1 deacetylation activity, while only phosphorylated Ser47 of SIRT1 induces ubiquitination of SIRT1 for degradation. For SIRT2, its phosphorylation at Ser327, Ser331, Ser335, Ser368, Ser372, and Ser473, notably represses its catalytic activity on histones with different kinases [186–189]. SIRT6 phosphorylation only affects its protein stability on Ser338, chromatin binding on Thr294 and mono-ADP ribosylation activity on Ser10 [190–192]. And until now, there is no evidence for SIRT7 phosphorylation.

It is worth noting that several enzymes, including EZH2, P300, KAT7, KAT13A, and almost of HDACs, own a relatively large number of functional phosphorylation sites in the summarized tables. EZH2 is comprised of SANT domain, CXC domain, and catalytic SET domain. The phosphorylated sites in the N-terminal domain of EZH2 by various protein kinases not only inhibits its methylation activity, but also promotes its ubiquitination for degradation. Only one site, Thr350, can be phosphorylated and activates enzymatic activity of EZH2. And phosphorylation of Ser646 in the region of SET domain of EZH2 promotes its degradation in malignant lymphoma [89]. Even it is reported that CXC domain of EZH2 is conformational flexible and structurally block SET domain for substrate binding, there is still unknown whether phosphorylation of these sites is dependent on the dynamic conformational plasticity to regulate EZH2 enzymatic activity or not [193]. Most phosphorylated sites in C-terminal of P300, ranging from 1830 to 2370 amino acids of P300 protein sequence, have been evidenced to enhance its acetylation activity, while there is complex for sites in the N-terminus of P300. Phosphorylation of Ser12 by MAPK, activates P300 catalytic activity in cancers, and phosphorylated Ser106 can stabilize P300 in response to DNA damage. It is well studied that Ser89 of P300 is phosphorylated by different kinases with diverse biological functions. AMPK can phosphorylate Ser89 of P300 to repress its acetylation activity, and the interactions between P300 and chromatin/proteins is determined by PKC/SIK2-mediated phosphorylation of P300 on Ser89. However, it is still confusing that how different kinases phosphorylate the same site of P300 and what kinds of changes on P300 itself lead to obvious distinctions of these functions. Therefore, the interaction network on these questions should be further studied. Similar with P300, HDACs phosphorylation has also been questioned. It has been found that Ser 424 of HDAC3 can be phosphorylated by five kinases, including CK2, TBK1, LRRK2, C-JUN, AND PINK1. All of these kinases enhance the deacetylation activity of HDAC3 by phosphorylating Ser424, while it is verified with experiments in distinct disease models, such as triple-negative breast cancer, Parkinson’s disease, and innate antiviral immunity. Combined with these single studies, it is complicated to some extent and there are no explanations on their internal relations.

**Table 4:** Phosphorylation of SIRT family proteins and its effect on the enzymatic activity

Isoforms of KMTs	Target residues	Kinases	Functional regulation upon phosphorylation	Involved in diseases and signaling pathways	References
SIRT1	Ser27	CaMKKβ	Stabilization	Inflammation	[194]
		PKC	DNA binding	Oligodendrocytes survival	[195]
		JNK1	Activation of enzymatic activity	Stress protection pathway	[196]
	Ser47	CaMKKβ	Stabilization	Inflammation	[194]
		CDK5	Stabilization	Diabetic Nephropathy	[197]
		p38 MAPK	Stabilization	Age-related diseases	[198]
		JNK/SAPK			
		JNK1	Activation of enzymatic activity	Obesity	[199]
		mTOR	Inhibition of enzymatic activity	DNA damage	[185]
	Ser164	CK2	Inhibition of its nuclear localization	Obesity	[200]
	Ser280	JAK1	Enhance of its interaction with STAT3	JAK1-STAT3 pathway	[201]
	Ser301				
	Thr344	AMPK	Inhibition of enzymatic activity	Liver cancer	[202]
	Thr522	DYRK1A/DYRK3	Activation of enzymatic activity	Cellular stress response	[203]
	Thr530	DYRK2	DNA binding		[204]
		AMPK	Enhance of its interaction with PABP1	Eukaryotic Poly(A)RNA Transport	[205]
	Ser615	LKB1	Enhance of intramolecular interactions in Sirt1	Mitochondrial metabolism	[206]
	Ser649	CK2	Activation of enzymatic activity	Alzheimer’s disease	[207]
	Ser659	CK2	Enhance of its interaction with HIC1	DNA damage	[208]
	Ser661	CK2			
	Ser669	LKB1	Enhance of intramolecular interactions in Sirt1	Mitochondrial metabolism	[206]
	Ser682	HIPK2	Inhibition of enzymatic activity	DNA damage	[209]
	Thr719	AMPK	Enhance of its interaction with PABP1	Eukaryotic Poly(A)RNA Transport	[205]
	Ser732	LKB1	Enhance of intramolecular interactions in Sirt1	Mitochondrial metabolism	[206]
SIRT2	Tyr104	c-SRC	Inhibition of enzymatic activity		[210]
	Ser327	GSK3β	Inhibition of enzymatic activity	Parkinson’s disease	[186]
	Ser335				
	Ser331	GSK3β			
		CDK2	Inhibition of enzymatic activity	Cell cycle	[187]
	Ser368	CDK1	DNA binding	Cell cycle	[211]
		CDK5	Inhibition of enzymatic activity	Cell cycle	[188]
	Ser372				
	Ser473	CK2	Inhibition of enzymatic activity	Dietary restriction (DR)-mediated lifespan extension	[189]
SIRT6	Ser10	JNK	DNA binding	DNA damage	[192]
	Ser338	AKT	Degradation	Breast cancer	[190]
		CSNK2A1	DNA binding	Osteosarcoma	[212]
	Thr294	PKC	Enrichment on chromatin	Colon Cancer	[191]

## Conclusions and perspectives

**Fig. 3:**
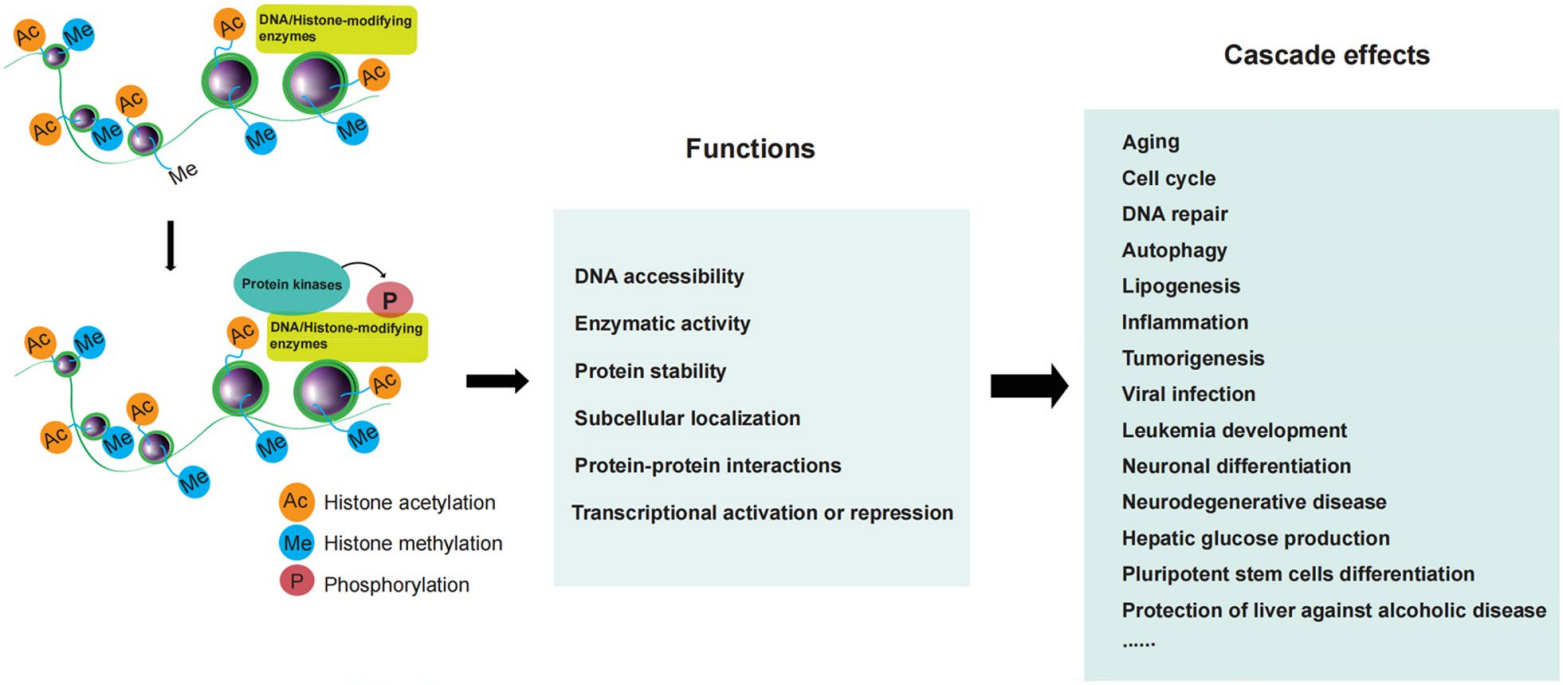
The model of transcriptional regulation of DNA/histone-modifying enzymes by phosphorylation

In this review, we specifically discuss how phosphorylation regulates two kinds of transcription cofactors, including DNA methylation/demethylation-related enzymes and histone-modifying enzymes (the model in Fig. 3). Many transcription factors occur transcriptional activation or repression via phosphorylation-enhanced enzymatic activity or phosphorylation-inhibited enzymatic activity. And also, phosphorylation controls the protein stability of TFs, nuclear localization, and the interaction with chromatin or other proteins. Precise mechanisms by which phosphorylation enhances or represses the transcriptional activity of these two kinds of enzymes are still poorly understood. Maybe it occurs either: (i) by a converted structural conformation that covers or uncovers a docking site for histones, (ii) by the removal of an inhibitory protein or molecule resulted from phosphorylation, (iii) by directly blocking the histone binding sites in the catalytic domain of TFs, (iv) by recruiting other proteins to tighten or relax chromatin.

Since the discovery of histone PTMs, it has been evidenced to be dynamic and reversible [213]. DNA and histone modifications occur through “writer” enzymes, including DNMTs, KMTs, and KATs. The opposite enzymes are known as “erasers” for eliminating histone modifications, which contain TETs, KDMs, HDACs. Other enzymes, some of which are called “readers”, can recognize and bind specifically modified histones and possibly interact with “writers” or “erasers”. DNA and histone modifications significantly affect chromatin structure and transcription regulation of genes. The phosphorylation of histone-modifying enzymes has opened up a new perspective for molecular mechanisms of transcription regulation. In general, phosphorylation-dependent enzymatic activation or inactivation of TFs, and even phosphorylation-mediated protein degradation or dissociation from chromatin, usually lead to aberrant gene expression and this effect is likely to be more severe in diseases, including cancers and mental disorders. In our review, we summarized all studied phosphorylated sites of DNA/histone-modifying enzymes described in previous researches. Interestingly, one serine residue (or threonine and tyrosine residue), which plays a crucial role in TFs functions, is reported to be phosphorylated by distinct kinases and regulates different genes’ expression during multiple subcellular processes, including cell differentiation, DNA damage and repair, inflammation, and metabolism. Substrate specificity is characterized by a protein kinase because the phosphorylation of a protein is determined by a targeted phospho-acceptor site in a consensus motif of a substrate [214]. However, one or two sites or motif cannot completely determine substrate specificity. Indeed, the interaction between TFs and protein kinases is also important for efficient substrate phosphorylation. Moreover, a specific protein kinase-dependent phosphorylation of TFs is also verified in one type of cell line. It is not clear whether different kinases compete for binding to one TFs and how to phosphorylate TFs at one site in an orderly fashion. And how different cell types influence TFs phosphorylation. Maybe phosphorylation involves TFs in the different signaling pathways which corporately regulate their cellular functions. These questions are still unresolved, so the further detailed study is needed to uncover the network of the known molecular mechanisms. Therefore, a comprehensive and systematic study on TFs phosphorylation will possibly expand our understanding of the transcriptional regulation of these DNA/histone-modifying enzymes via new advanced technologies. In addition, deletion of specific kinases that target one transcription factor, or insertion of mutations of phosphorylated sites on transcription factors in a mouse model, will enhance our comprehension and genetically prove the importance of phosphorylation in regulating themselves and overall functions of TFs.

## Data Availability

Not applicable.

## Abbreviations

5caC: 5-Carboxylcytosine
5fC: 5-Formylcytosine
5hmC: 5-Hydroxymethylcytosine
5mC: 5-Methylcytosine
ADA3: Alteration/Deficiency in Activation 3
AdoMet: S-adenosyl-methionine
AMPK: MP-activated kinase
AMPK: (AMP)-activated protein kinase
ATM: Ataxia-telangiectasia mutate
BER: Base excision repair
CaMKII: Calmodulin kinase II
CDK1: Cyclin-dependent kinase 1
CDK5: Cyclin-dependent kinase 5
CDKs: Cyclin-dependent kinases
CK2: Casein kinase 2
DSBs: Double-strand breaks
EGFR: Epidermal Growth Factor Receptor
EKLF: Kruppel-like factor
ERK1/2: Extracellular signal-regulated kinase 1/2
ERK2: Extracellular signal-regulated kinase 2
ETS: E-twenty-six
EZH2: Zeste homolog 2
FGFR3: Fibroblast growth factor receptor 3
H3K4: Histone H3 lysine 4
HATs: Histone acetyltransferases
HDACs: Histone deacetylases
HGP: Hepatic glucose production
HIPK2: Homeodomain-interacting protein kinase 2
HMGA2: High-mobility group AT-hook 2
HR: Homologous recombination
HSC: Hematopoietic stem cell
JNK: C-Jun N-terminal kinase
KDMs: Histone lysine demethylases
KIF5: Kruppel-like factor 5
KLF4: Kruppel like factor 4
KMTs: Lysine methyltransferases
LRRK2: Leucine-rich repeat kinase 2
MK: Megakaryocyte
MLL1: Mixed lineage leukemia 1
MST1: Mammalian sterile 20-like kinase 1
MYHC1: Myosin heavy chain 1
NFATc1: Nuclear factor of activated T-cells c1
NLK: Nemo-like kinase
NLS: Nuclear localization signal
PcG: Polycomb group proteins
PDK1: Protein kinase D1
PGM1: Phosphoglucomutase 1
PIK1: Polo-like kinase 1
PINK1: PTEN-induced putative kinase 1
PKA: Protein kinase A
PKB: Protein kinase B
PKC: Protein kinase C
PKD: Protein kinase D
PRMTs: Protein arginine methyl transferases 1
PTMs: Post-translational modifications
RAR: Retinoic acid receptor
RPA: Replication protein A
Ser: Serine
TBK1: TANK-binding kinase
TDG: Thymine-DNA glycosylase
TFs: Transcriptional factors
Thr: Threonine
Tyr: Tyrosine
